# A Cross-Sectional Study to Evaluate the Effects of Age and Duration of HIV Infection on Anxiety and Depression in Cisgender Men

**DOI:** 10.1007/s10461-021-03373-y

**Published:** 2021-07-21

**Authors:** Sara Gianella, Rowan Saloner, Genevieve Curtin, Susan J. Little, Anne Heaton, Jessica L. Montoya, Scott L. Letendre, María J. Marquine, Dilip V. Jeste, David J. Moore

**Affiliations:** 1 Division of Infectious Diseases and Global Public Health, Department of Medicine, University of California San Diego, 9500 Gilman Drive MC 0679, La Jolla, CA 92093-0679 USA; 2 Division of Geriatrics, Gerontology and Palliative Care, University of California, La Jolla, USA; 3 Department of Psychiatry, University of California San Diego, La Jolla, USA; 4 Sam and Rose Stein Institute for Research on Aging, University of California San Diego, La Jolla, USA; 5 Department of Neurosciences, University of California San Diego, La Jolla, USA

**Keywords:** HIV, Anxiety, Depression, Duration of HIV infection

## Abstract

This observational cross-sectional study of 152 people with HIV (PWH) examined the effects of age and estimated duration of HIV infection (EDI) on depressive and anxiety symptoms. All participants were cisgender men and completed the Profile of Moods State (POMS), a self-report inventory of current (i.e., past week) mood states. Overall, study results confirmed higher levels of anxiety and depression in PWH compared to individuals without HIV. Age group (< 50 or ≥ 50 years) moderated the effect of EDI (< 3 or ≥ 3 years) on mood disturbance. Specifically, younger PWH with early diagnosed infection exhibited the highest levels of depression and anxiety, whereas depression and anxiety were attenuated in older PWH with early infection such that their POMS scores did not significantly differ from the HIV-negative and chronically HIV-infected groups. Despite the small sample size and other important limitations in our study design, our preliminary findings confirm previous observations that older people may have some adaptive ability to better handle the acute psychological stressors associated with recent HIV infection.

## Introduction

Depression is a leading cause of disability worldwide [1] and is more common in people with HIV (PWH) [2–7] than in people without HIV [8]. Nearly one half of PWH experience one or more psychiatric disorders in their lifetime [6]. Specifically, PWH are two to three times more likely to experience generalized anxiety disorder, panic disorder, major depressive disorder (MDD), or dysthymic disorder [6, 9] than persons without HIV. While stigma largely contributes to these findings, the physical effects of HIV and antiretroviral therapy (ART) might also play a role [10, 11]. Notably, PWH experiencing a psychiatric disorder are more likely to have poor adherence to medication [12]. Similarly, those with suboptimal adherence are approximately three times more likely to have moderate to severe symptoms of depression than those reporting optimal adherence [13]. Poor medication adherence can lead to increased drug resistance [14] and is a major determinant of virologic failure, HIV disease progression, hospitalization, mortality, and health care costs [15], which can all in turn contribute to psychological distress. Importantly, for PWH, experiencing a psychiatric disorder has a significant impact on health-related quality of life [4, 12]. For all these reasons, further exploration of the connection between HIV and psychiatric disorders remains vital, especially as life expectancy for PWH has increased considerably over the last two decades [16]. Early exploratory work has indicated that older PWH are more likely to report suicidal ideation [17] and experience social isolation [18] compared to younger PWH and seronegative persons. On the other hand, studies have indicated that prevalence of many psychiatric disorders (e.g., anxiety) decreases slightly with age among the general population [19] and that older PWH may not be at disproportionate risk for anxiety relative to younger PWH [20–22].

In fact, whereas older people are more likely to have additional medical co-morbidities, they may also be better equipped to handle adverse events and overall tend to be more resilient and exhibit other positive psychological traits [23, 24]. For example, older persons are more likely to disengage from stressful situations compared to their younger counterparts, reducing negative emotional effects [25]. Older adults also experience and process emotions differently than younger adults, with less bias towards negative emotion and less autonomic response [26–28]. Thus, older age represents both a risk and protective factor for anxiety among PWH [20].

Importantly, most previous studies characterizing medical and psychiatric comorbidities among PWH have only included chronically infected older PWH and thus the effects of duration of infection and age have not been adequately investigated. In the present cross-sectional study, we aimed to examine the differential effects of age and duration of HIV infection on depression and anxiety among PWH.

## Methods

### Study Participants

Participants were 90 men, cisgender with HIV and 62 men of comparable demographics without HIV (HIV^−^) prospectively enrolled in the Multi-Dimensional Successful Aging Among HIV-infected Adults study at the University of California San Diego between 2013 and 2018 [29]. PWH were recruited from HIV-focused community clinics and health care providers in the San Diego area. For this cross-sectional study, we recruited additional older and younger individuals with acute and early HIV infection from the Primary Infection Research Consortium (PIRC) [30, 31] and from the community. Control participants without HIV were demographically matched and recruited through UCSD-based aging programs, local flyers, and presentations as well as from our previous research pool of comparison participants. Inclusion criteria (for both groups) were: capable of giving informed consent, and being > 18 year old. PWH were required to take antiretroviral therapy and have virologic suppression. Exclusion criteria (for both groups) were: presence of an existing neurological condition unrelated to HIV, hepatitis C virus, positive breathalyzer test for alcohol or urine toxicology for illicit drugs (except marijuana) on the day of assessment, and diagnosis of a psychotic disorder. Women were excluded from analysis due to insufficient numbers across study groups (only one woman was enrolled in the older/recently infected group). The study was reviewed and approved by the University of California San Diego Human Research Protections Program. All participants provided written informed consent.

### Neurobehavioral Evaluation

All participants completed the Profile of Moods State (POMS), a 65-item self-report questionnaire of current mood states (i.e., symptoms within the past week) [32]. Each item asks respondents to rate past week feelings (e.g., “anxious”) on a five-point Likert-type scale, ranging from 0 (“not at all”) to 4 (extremely). The POMS has demonstrated strong internal reliability and predictive validity for clinical mood disorder diagnoses among PWH [33, 34]. The Depression/Dejection subscale consists of 15 items (score range 0–60) reflecting depressive symptoms and the Tension/Anxiety subscale consists of 9 items (score range 0–36) reflecting anxiety symptoms. The Depression/Dejection and Tension/Anxiety subscales were used as primary outcomes in the present analysis. In addition to the POMS, participants were administered the Composite International Diagnostic Interview (CIDI) [35]. The CIDI is a structured, lay-administered, computer-based interview that was used to assess for diagnoses of current and lifetime substance use disorders and MDD based on Diagnostic and Statistical Manual of Mental Disorders—Fourth Edition (DSM-IV) criteria.

Participants also underwent a comprehensive neurocognitive assessment that evaluated seven neurocognitive domains commonly impacted by HIV [36, 37]. Test scores were adjusted for known demographic influences (i.e., age, education, sex, and race) on neurocognitive performance [38–40]. Deficit scores that preferentially weight impaired over normal performance were calculated for each domain and averaged across domains to compute a global deficit score (GDS). Consistent with prior studies, neurocognitive impairment (NCI) was classified using a validated cut-point of GDS ≥ 0.5 [41].

### Neuromedical Evaluation

Participants underwent a comprehensive neuromedical history evaluation assessing for HIV-specific characteristics and comorbid medical conditions (e.g., hypertension, diabetes). All participants were tested for HIV status using an HIV/HCV finger stick point-of-care test (Miriad, MedMira, Nova Scotia, Canada). For participants with confirmed HIV disease, specific HIV treatment and disease characteristics were evaluated via structured clinical interview (i.e., AIDS history, nadir CD4^+^ count, estimated duration of infection, ART regimen) and laboratory assessment (i.e., current CD4^+^ T-cell count, HIV RNA in plasma). HIV viral load in plasma was measured using reverse transcriptase-polymerase chain reaction (Amplicor; Roche Diagnostics, Indianapolis, IN). Participants were considered undetectable at viral loads below the lower limit of quantitation of 50 copies/ml.

### Statistical Analysis

For the present study, participants were stratified a priori by age [younger (< 50 years) or older (≥ 50 years)] and estimated duration of HIV infection (EDI) [HIV^−^, chronic (≥ 3 years) or recently infected (< 3 years)], resulting in six study groups: HIV^−^/Younger (n = 30), HIV^−^/Older (n = 32), Chronic/Younger (n = 26), Chronic/Older (n = 35), Recently infected/Younger (n = 19), and Recently infected/Older (n = 10).

Age and EDI group differences on background characteristics (i.e., demographics, neuropsychiatric, cardiometabolic, and HIV disease characteristics) were examined using analysis of variance (ANOVA), Wilcoxon/Kruskal–Wallis tests, and Chi-square statistics as appropriate. To determine the interactive effects of age and EDI on symptoms of depression and anxiety, two-way ANOVA (age x EDI) separately modeled POMS Tension/Anxiety and Depression/Dejection subscale scores. Following interpretation of interaction effects, a priori planned comparisons examined pairwise effects of EDI group within each age group, as well as the effect of age group within each EDI group. Pairwise comparisons were conducted using the Wilcoxon rank-sum test and estimates of effect size for these tests were calculated using r statistics for non-parametric group comparisons (${\text{r}} = {\text{Z}}/\sqrt {\text{n}}$). Small effect sizes range from r = 0.1–0.3, medium from r = 0.3–0.5, and large from r > 0.5. Given constraints in statistical power due to small age × EDI group sizes, analyses were not adjusted for multiple comparisons and are therefore considered preliminary. To confirm independent contributions of age and EDI on POMS subscale scores, ANCOVA models considered covariates that differed by age or EDI group at *p*-value < 0.10 (i.e., race/ethnicity, lifetime MDD, lifetime substance use disorder, hypertension, hyperlipidemia) and years of education given its potential association with mood [42–44]. Covariates were retained in the final ANCOVA based on backward model selection guided by Akaike information criteria (AIC). All analyses were conducted using JMP Pro version 14.0.0 (JMP®, Version < 12.0.1 > . SAS Institute Inc., Cary, NC, 2018).

## Results

### Participant Characteristics

Table 1 summarizes the characteristics of the study cohort by EDI and age groups. There were no significant EDI group differences in age within older participants (mean age range: 57–60 years); however, among younger participants, the recently infected group [mean (standard error) = 36 (1.2) years] was significantly younger than the HIV^−^ [mean (SE) = 44 (0.9) years] and chronic groups [mean (SE) = 43 (1.0) years]. Groups did not differ with respect to years of formal education. Trend-level differences in race/ethnicity were observed across both age and EDI groups, with the recently infected and younger groups having the highest proportion of racial/ethnic minorities. The recently infected and chronic groups did not significantly differ with respect to rates of neurocognitive impairment, yet both PWH groups had higher rates of lifetime MDD than the HIV^−^ group, and the chronic HIV group had higher rates of a lifetime substance use disorder than the recently infected PWH group and HIV^−^ group. With respect to cardiometabolic conditions, rates of hypertension and hyperlipidemia were significantly higher in the chronic group, compared to HIV^−^ and recently infected groups, and rates were significantly higher in the older group compared to the younger group. As expected, the chronic/older group had the longest EDI (median = 25 years), followed by the chronic/younger group (median = 13 years), then the recently infected groups (median = 2 years). Although chronically infected participants had significantly lower nadir CD4^+^ T cell counts, higher rates of lifetime AIDS diagnoses, and longer estimated days to first initiation of ART than recently infected participants, EDI groups did not differ with respect to current CD4^+^ T cell counts.

**Table 1:** Demographic and clinical characteristics by age and EDI group

Variable	HIV^−^		Chronic		Recent			
	Older (n = 32)	Younger (n = 30)	Older (n = 35)	Younger (n = 26)	Older (n = 10)	Younger (n = 19)	*p1*^a^	*p2*^b^
Demographics								
Age (years), mean (SD)	57.1 (4.56)	44.0 (3.90)	57.0 (4.43)	43.3 (4.53)	59.9 (5.72)	36.4 (7.91)		
Education (years), mean (SD)	14.7 (2.07)	15.4 (2.33)	15 (2.37)	14.5 (2.37)	15.1 (3.73)	14.7 (2.64)	0.915	0.926
Race/Ethnicity							0.094	0.063
Non-Hispanic White, n (%)	25 (78.1)	19 (63.3)	23 (65.7)	14 (53.9)	6 (60)	8 (42.1)		
Hispanic, n (%)	4 (12.5)	6 (20)	5 (14.3)	8 (30.8)	3 (30)	8 (42.1)		
Black, n (%)	3 (9.4)	4 (13.3)	5 (14.3)	2 (7.7)	1 (10)	0 (0)		
Other, n (%)	0 (0)	1 (3.3)	2 (5.7)	2 (7.7)	0 (0)	3 (15.8)		
Neuropsychiatric Characteristics								
Neurocognitive impairment, n (%)	6 (18.8)	8 (26.7)	16 (45.7)	7 (26.9)	4 (40)	7 (36.8)	0.134	0.557
Lifetime MDD, n (%)	6 (18.8)	4 (13.3)	17 (50)	12 (46.2)	3 (30)	7 (36.8)	< 0.001	0.642
Lifetime SUD, n (%)	17 (53.1)	8 (26.7)	20 (58.8)	19 (73.1)	3 (30)	10 (52.6)	0.018	0.685
Cardiometabolic Conditions								
BMI (kg/m^2^), mean (SD)	27.7 (3.68)	27.2 (3.63)	27.3 (4.66)	28.9 (5.9)	27 (5.51)	27.1 (4.15)	0.693	0.625
Hypertension, n (%)	9 (28.1)	2 (6.7)	19 (54.3)	8 (30.8)	3 (30)	3 (15.8)	0.003	0.002
Hyperlipidemia, n (%)	12 (37.5)	3 (10)	26 (74.3)	4 (15.4)	3 (30)	2 (10.5)	0.002	< 0.001
Diabetes, n (%)	2 (6.3)	1 (3.3)	4 (11.4)	4 (15.4)	3 (30)	0 (0)	0.256	0.281
HIV Disease Characteristics								
Duration of HIV infection (years), median [IQR]			25.1 [17.5, 26.6]	12.6 [5.4, 22.1]	1.5 [0.5, 2.4]	1.6 [0.8, 2.2]	< 0.001	< 0.001
AIDS diagnosis, n (%)			20 (57.1)	16 (61.5)	1 (10)	3 (15.8)	< .001	0.671
Nadir CD4 count (cells/mm^3^), median [IQR]			198 [59, 350]	161 [45, 299]	436 [315, 585]	423 [295, 597]	< .001	0.961
Current CD4 count (cells/mm^3^), median [IQR]			638 [485, 888]	603 [410, 770]	591 [348, 803]	717 [486, 902]	0.920	0.616
Days prior to ART initiation, n (%)			159 [30, 1706]	350.5 [15, 715]	61 [30, 91]	12 [0, 51]	0.002	0.108

*ART* antiretroviral therapy, *BMI* body mass index, *MDD* Major Depressive Disorder, *SUD* substance use disorder

^a^*p*-value #1 represents EDI group comparisons

^b^*p*-value #2 represents age group comparisons

### Age, EDI and Anxiety

**Fig. 1:**
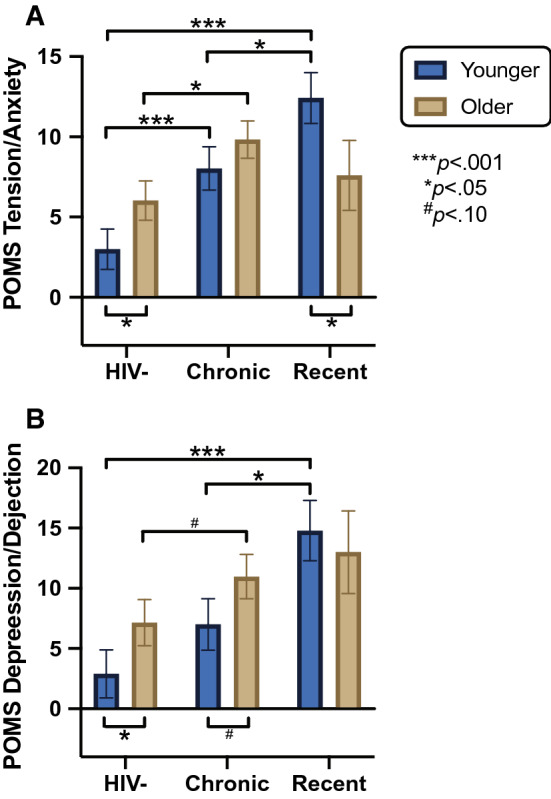
Data are expressed as mean +/− standard error. Significant or trend-level age group differences, stratified by estimated duration of infection (EDI), are displayed below the x-axis. Significant or trend-level EDI group differences, stratified by age, are displayed above the x-axis. Statistical significance determined with Wilcoxon pairwise comparisons

Table 2 and Fig. 1 present age by EDI group differences in POMS anxiety and depression scores. Univariably, anxiety scores were significantly higher in both PWH groups (recent and chronic EDI) as compared to participants without HIV (*p*s < 0.003) but did not differ by age group (*p* = 0.359). In the two-way ANOVA, a significant interaction was detected between age and EDI on anxiety scores (*F*_2_,_146_ = 3.10, *p* = 0.048, $\eta_{p}^{2} = 0.04$). Planned pairwise comparisons revealed that older age significantly related to higher anxiety scores in HIV^−^ participants (*p* = 0.014), lower anxiety scores in recently infected PWH (*p* = 0.306) and did not significantly predict anxiety scores in chronically infected PWH (*p* = 0.045). Notably, recently infected PWH had significantly higher anxiety scores than chronically infected PWH and HIV^−^ participants only within the younger group (*p*s < 0.024).

**Table 2:** POMS anxiety and depression score comparisons by age and EDI group

POMS Score	HIV^−^		Chronic		Recent	
	Older (n = 32)	Younger (n = 30)	Older (n = 35)	Younger (n = 26)	Older (n = 10)	Younger (n = 19)
Anxiety	6.0 (1.2)	3.0 (0.7)	9.8 (1.3)	8.0 (1.5)	7.6 (3.0)	12.4 (1.7)
Depression	7.2 (1.8)	2.9 (1.5)	11 (1.8)	7.0 (1.9)	13.0 (5.1)	14.8 (3.3)

	Anxiety			Depression		
	r	95% CI	*p*	r	95% CI	*p*
Pairwise age differences by EDI group						
HIV^−^						
Old vs. young	**0.31**	**0.06**, **0.56**	**0.014**	**0.28**	**0.03**, **0.53**	**0.028**
Chronic						
Old vs. young	0.13	− 0.11, 0.38	0.306	0.23	− 0.02, 0.49	0.067
Recent						
Old vs. young	− **0.37**	− **0.74**, − **0.01**	**0.045**	− 0.14	− 0.23, 0.50	0.462
Pairwise EDI differences by age group						
Old						
Chronic vs. HIV^−^	**0.45**	**0.19**, **0.71**	** < 0.001**	**0.24**	**0.00**, **0.48**	**0.048**
Recent vs. HIV^−^	0.02	− 0.28, 0.32	0.905	0.20	− 0.10, 0.50	0.190
Recent vs. chronic	− 0.20	− 0.49, 0.09	0.175	− 0.01	− 0.30, 0.28	0.935
Young						
Chronic vs. HIV^−^	**0.30**	**0.07**, **0.54**	**0.013**	0.26	− 0.01, 0.52	0.056
Recent vs. HIV^−^	**0.67**	**0.39**, **0.95**	** < 0.001**	**0.64**	**0.32**, **0.92**	** < 0.001**
Recent vs. chronic	**0.34**	**0.05**, **0.53**	**0.023**	**0.38**	**0.09**, **0.67**	**0.011**

Pairwise comparisons were conducted using Wilcoxon rank-sum test and effect size estimates were calculated as ${\text{r}} = {\text{Z}}/\sqrt {\text{n}}$. Effect sizes range from small (r = 0.1–0.3), medium (r = 0.3–0.5), and large (r > 0.5). Bolded items are significant at *p* < 0.05.

### Age, EDI and Depression

Univariably, depression scores were significantly higher in both PWH groups compared to HIV^−^ (*p*s < 0.003), as well as in the older group compared to the younger group (*p* = 0.045). In the two-way ANOVA, the age by EDI interaction did not significantly predict depression scores (*F*_2_,_146_ = 0.80, *p* = 0.451, $\eta_{p}^{2} = 0.01$). When testing for independent main effects, EDI group remained independently associated with depression scores (*F*_2_,_148_ = 7.79, *p* < 0.001, $\eta_{p}^{2} = 0.09$) while age group exhibited a trend-level association with depression scores (*F*_1_,_148_ = 2.96, *p* = 0.087, $\eta_{p}^{2} = 0.02$). Older age related to higher depression scores in HIV^−^ participants (*p* = 0.028), trended toward higher depression scores in chronically infected PWH (*p* = 0.067), and did not significantly predict depression scores in recently infected PWH (*p* = 0.462). Similar to the anxiety analysis, recently infected PWH had significantly higher depression scores than chronically infected PWH and HIV^−^ participants only within the younger group (*p*s < 0.012).

### ANCOVA Models

The age × EDI interaction on anxiety scores persisted in ANCOVA (*p* = 0.043), with only a lifetime diagnosis of MDD additionally relating to higher anxiety scores [b (SE) = 2.95 (1.24), *p* = 0.019]. The independent effect of EDI group on depression scores remained significant in ANCOVA (*p*s < 0.004), whereas the independent effect of age group was weakened (*p* = 0.220). With respect to covariates, lifetime MDD [b (SE) = 5.82 (1.94), *p* = 0.003], lifetime substance use disorder [b (SE) = 3.88 (1.77), *p* = 0.030], years of education [b (SE) = 0.65 (0.36), *p* = 0.073], hypertension [b (SE) =  − 4.02 (2.16), *p* = 0.065], and hyperlipidemia [b (SE) = 3.94 (2.20), *p* = 0.076] were retained (by AIC criteria) as predictors of higher depression scores.

## Discussion

Previous research has demonstrated higher levels of mental health disorders in PWH, when compared to the the general population [6, 9, 45]. These mental health disorders can decrease health-related quality of life and medication adherence, leading to worse outcomes for PWH [12, 15]. As the PWH population ages, it is especially important to consider the intersection between HIV status, age, and mental health. While this is an area that has been explored previously, EDI has not been adequately considered. Thus, as part of this cross-sectional study, we evaluated the effects of age and EDI on self-reported depression and anxiety symptoms. Despite the small sample size and other important limitations in our study design, we confirmed that PWH experience higher levels of symptoms of anxiety and depression when compared to HIV^−^ individuals [6, 9, 45]. We also found overall higher levels of depression in older individuals, as compared to younger individuals. Levels of anxiety, on the other hand, did not differ by age groups among PWH.

When analyzing the anxiety data by HIV and EDI status, we found higher levels of anxiety in younger individuals with recently diagnosed HIV infection, when compared to their older counterparts, but no age-difference among individuals with chronic HIV. Conversely, within the HIV^−^ group, anxiety was higher among older compared to younger individuals. This pattern of results produced a significant age by group interaction, which remained unaltered after adjustment for lifetime MDD and nadir and current CD4 counts, thereby highlighting the strength of our findings.

Within the younger group, we found higher depression scores among recently infected PWH, as compared to chronically infected PWH and HIV^−^. Conversely, within the older group, we found a greater level of depression among individuals with chronic HIV, as compared to HIV^−^ individuals. Finally, older individuals had significantly more depression than younger within the HIV^−^ group. Although the magnitude of these results was not large enough to produce a significant interaction effect given the small sample sizes, depression scores remained significantly higher among younger recently infected PWH after adjustment for other relevant factors. Conversely, the independent effect of age was weakened after adjustment for covariates (which included relevant age-related co-morbidities).

Overall, our findings suggest that older PWH may have some adaptive ability to better handle more recent or acute adverse events. Another recent paper [46] also found increased anxiety and depression in early HIV infection but did not enroll older adults and therefore could not examine the impact of older age. Our study importantly extends the literature in this way.

It has previously been demonstrated that older adults have diminished cardiovascular response when discussing emotional conflicts, remember fewer negative words than positive, and have less activity in the amygdala when presented with negative pictures than younger adults, indicating that older people are less stimulated by negative information than their younger counterparts [47–49]. This is consistent with other studies reporting increased anxiety in younger PWH [21, 22, 45].

The limitations of this study include the cross-sectional study design, the small sample size, and male only participants. While the older and younger groups with early HIV infection were recruited specifically for this cross-sectional study, because of budgetary limitations we were limited in the number of participants to be included in this analysis. Also, our older group covers a wide range of years and does include people from the higher end of middle age as well as older people, which might be different. The younger recently infected participants are substantially different in age from the chronic younger group, and the duration of HIV status varies widely in the chronic group whereas the variability is quite small in the recent group. Further, while the HIV-negative comparison group was recruited from the broader community to be demographically comparable, it was not risk-matched to the PWH, and thus our comparisons might be confounded by some factors other than their HIV status.

Also, the recently infected group of PWH was drawn from a different cohort than the chronic group, which might introduce some additional selection bias. Also, how age impacts the experience of a PWH likely depends on when in the history of the HIV/AIDS epidemic they were diagnosed. Specifically, there is likely to be a difference in experience between being diagnosed as HIV positive more recently compared to being diagnosed in the 1980s. As such, duration of HIV status is confounded with these cultural and historical shifts.

Nevertheless, this study is novel in its attempt to explore the combined effects of age, HIV status, and duration of infection on mental health outcomes. Future larger studies should be performed to expand these findings and identify underlying mechanisms and risk factors, which might differ by age and EDI. This could serve as the basis to design future interventions, as for example peer-to-peer intervention of older adults sharing their wisdom with younger PWH.
